# Abomasitis in calves: A retrospective cohort study of 23 cases (2006‐2016)

**DOI:** 10.1111/jvim.15726

**Published:** 2020-02-14

**Authors:** Eloi Guarnieri, Gilles Fecteau, Julie Berman, André Desrochers, Marie Babkine, Sylvain Nichols, David Francoz

**Affiliations:** ^1^ Faculty of Veterinary Medicine Université de Montréal Saint‐Hyacinthe Québec Canada

**Keywords:** abomasum, bloat, cattle, *Clostridium* spp., *Escherichia coli*, tympany

## Abstract

**Background:**

Abomasitis is a syndrome affecting young milk‐fed calves. The current veterinary literature describes mainly its necropsy findings.

**Objectives:**

To describe the clinical presentation, complementary tests, treatments, and case‐fatality rate of calves with a clinical diagnosis of abomasitis and to identify potential factors associated with outcome.

**Methods:**

Observational retrospective cohort study (2006‐2016). Review of the medical records of calves <3 months of age presented with abdominal and abomasal distension for <7 days that were clinically diagnosed with abomasitis at the Faculty of Veterinary Medicine of the Université de Montréal. A follow‐up examination was conducted by telephone interview.

**Animals:**

Twenty‐three calves clinically diagnosed with abomasitis.

**Results:**

Median age of presentation was 3 days (range, 0‐62 days). The typical duration of the clinical course was <24 hours (15/23). On admission, the 2 most common clinical signs were anorexia (13/14) and positive succussion (13/14). Hyper‐l‐lactatemia (15/16) and increased γ‐glutamyl‐transferase activity (13/14) were the most common laboratory abnormalities. Hypoproteinemia (19/22) and a left shift (15/18) of the neutrophils also were observed. The short‐term case‐fatality rate was 52% (12/23). The clinical diagnosis was confirmed on all necropsied calves. *Clostridium* spp. and *Escherichia coli* were the most frequently isolated bacteria. Based on univariate statistical analysis, the surviving calves were significantly (*P* < .05) less hypothermic, less acidemic, less hyper‐l‐lactatemic, and had lower serum creatinine concentrations on admission than did the deceased calves.

**Conclusions and Clinical Importance:**

In our study, abomasitis was associated with a guarded prognosis.

**Abbreviations**
Aero: aerobactin
FMV: Faculty of Veterinary Medicine
GGT: γ‐glutamyl‐transferase
GLDH: glutamate dehydrogenase
IF: immunofluorescence
P: fimbriae P
SAS: Statistical Analysis System
SBP: serum biochemistry profile
STa: thermostable enterotoxin a
Stx: Shiga toxin
TCO_2_: total carbon dioxide
TMS: trimethoprim‐sulfadoxine
Tsh: temperature‐sensitive hemagglutinin
VMTH: Veterinary Medical Teaching Hospital

## 1 INTRODUCTION

Abdominal distension had multiple causes in calves.^1^ These include primary diseases of the forestomachs, abomasum, or small and large intestine, as well as systemic diseases such as peritonitis. More specifically, the abomasum can be distended secondary to abomasitis syndrome, abomasal ulcers, paralyzing gastroenteritis, abomasal displacements, and volvulus.^2^, ^3^

Abomasitis is reported in the veterinary literature using different names: abomasal tympany, abomasal bloat, and braxy‐like disease.^2^, ^4^, ^5^, ^6^, ^7^ This syndrome appears to be multifactorial. Some predisposing factors are related to inadequate milk administration: large and unique milk feedings, cold milk, carbohydrate‐rich lacto replacer, esophageal tube feeding, or inadequate equipment hygiene.^2^, ^4^, ^5^, ^7^, ^8^ Possible bacterial pathogens incriminated include *Clostridium perfringens* type A,^7^, ^9^, ^10^, ^11^, ^12^ *Sarcina* spp.,^8^, ^13^ *Escherichia coli*,^13^ *Lactobacillus* spp.,^8^ and *Campylobacter* spp.^13^, ^14^

Abomasitis is reported to affect preferentially young milk‐fed calves. The veterinary literature on abomasitis documents single case reports or case series. The available clinical picture includes sudden death, lassitude, colic, diarrhea, and abdominal distension. The abomasum is distended and filled by fluid, gas, or some combination of these.^2^, ^5^, ^6^, ^7^, ^8^, ^9^, ^10^, ^11^, ^12^, ^14^, ^15^, ^16^, ^17^, ^18^, ^19^ Macroscopic and microscopic lesions observed during necropsy are well described in the veterinary literature. The abomasum is distended by a large amount of reddish‐brown, foul‐smelling liquid. Acute emphysematous necrotizing hemorrhagic inflammation of the abomasal mucosa is reported. The presence of abomasal ulcers is inconsistent. Submucosal lesions include edema, hemorrhage, and emphysema. The current veterinary literature on abomasitis consists of necropsy findings with few details on clinical presentation and treatment.

The main objective of our observational retrospective cohort study was to describe the signalment, clinical presentation, results of complementary tests, treatment, and short‐ and long‐term case‐fatality rate of calves <3 months of age presented to the Veterinary Medical Teaching Hospital (VMTH) of the Faculty of Veterinary Medicine (FMV) of the Université de Montréal between 2006 and 2016 and diagnosed with an abomasitis without left abomasum displacement or abomasal volvulus. Our secondary objective was to identify potential factors associated with outcome. Our hypotheses were that (1) most affected calves will have a poor prognosis, even if they receive intensive medical treatment, and that (2) hematologic and biochemical changes will be compatible with sepsis and upper gastrointestinal stasis.

## 2 MATERIALS AND METHODS

Our study was an observational retrospective cohort study.^20^ The Strengthening the Reporting of Observational Studies in Epidemiology Vet Statement (Veterinary Extension)^21^ was used to report this study (Data S1).

### 2.1 Definitions used

In our study, the term abomasitis was considered similar to the term abomasal tympanism. A clinical diagnosis of abomasitis was defined as a diagnosis of abomasitis made by the clinician, during the hospitalization, based on clinical findings and complementary tests. Excluded animals were those diagnosed with left abomasal displacement or abomasal volvulus. A surviving calf was defined as a patient discharged from the VMTH. A deceased calf was defined as 1 that died naturally or was euthanized during hospitalization. The short‐term case‐fatality rate was defined as the proportion of deceased calves during hospitalization. The long‐term case‐fatality rate was defined as the proportion of deceased calves during or after hospitalization.

### 2.2 Case selection

Calves with abomasal disease <3 months of age were selected. They were admitted to the VMTH of the FMV between January 1, 2006, and July 31, 2016. The inclusion criteria chosen were duration of clinical signs <7 days and abdominal and abomasal distension confirmed by ultrasonography, laparotomy, necropsy, or some combination of these. Finally, calves with a clinical diagnosis on admission of left abomasum displacement or abomasal volvulus were excluded.

### 2.3 Data collection

Information about signalment, clinical signs, serum biochemistry profile (SBP), and CBC within the first 24 hours after admission, treatments during hospitalization, and bacteriological and necropsy results were collected retrospectively when available.

Point‐of‐care blood testing upon admission (microhematocrit; manual refractometry; blood gas analysis by using the ABL80 Flex Co‐ox, Radiometer America, Brea, California; blood l‐lactate concentration using the Lactate Pro, Arkray Factory Inc., Shiga, Japan; and blood glucose concentration using the glucometer, Breeze 2, Ascensia Diabetes Care, Basel, Switzerland ) was chosen when available for statistical analysis instead of delayed results from our medical laboratory (hematology: Advia 120, Siemens Healthineers, Erlangen, Germany; serum biochemistry: Unicel DxC 600, Beckman Coulter, Brea, California).

Follow‐up examinations by phone were carried out in February 2018 by a single investigator. The owners of the calves were asked about long‐term survival of the calves and, if they died, the time between discharge and death. Three attempts were made to reach the owner; otherwise, the data were declared missing.

### 2.4 Statistical analysis

A descriptive statistical analysis was performed. Regardless of their distributions, quantitative variables were characterized by means, SDs, medians, and ranges. Qualitative variables were characterized by frequency of occurrence, with the denominators depending on the amount of data available.

Individuals were grouped, and comparisons between surviving calves and deceased calves were conducted with respect to short‐ and long‐term outcomes. Univariate statistical analyses were performed to identify variables associated with outcome. The exact chi‐squared test was used to evaluate the association between the binary variables of the 2 groups. The binary variables were decubitus, lassitude, mucosal color, ping, succussion, abnormal feces, anorexia, suckling reflex, colic, and toxic changes. In view of the small numbers and large variations observed, the unequal variances *t* test was used to evaluate the statistical association between the continuous variables of the 2 groups. The continuous variables were rectal temperature, heart rate, respiratory rate, blood pH, serum sodium, potassium and chloride concentrations, total carbon dioxide (TCO_2_) concentration, blood glucose concentration, l‐lactate concentration, azotemia as assessed by serum creatinine concentration, PCV, total solids, serum fibrinogen concentration, leukocyte, segmented neutrophil, and immature neutrophil counts, and γ‐glutamyl transferase (GGT) and glutamate dehydrogenase (GLDH) activities. Some variables were transformed using the base 10 logarithm to normalize the distributions before statistical analyses: serum creatinine concentration, leukocyte count, segmented neutrophil count, and GGT and GLDH activities. The Cochran‐Mantel‐Haenszel test was used to evaluate the statistical association between the 2 groups for the variables with several categories, including evolution time and dehydration. The nonparametric Wilcoxon test was used to evaluate the statistical association between the 2 groups for variables having a non‐normal distribution, including age on admission and hospital stay. Statistical analyses were performed using Statistical Analysis System (SAS) analysis software version 9.3 (SAS Institute Inc., Cary, North Carolina). The significance threshold used was 0.05. Missing data were not considered in these statistical analyses.

## 3 RESULTS

Of the 1801 calves <3 months of age that were admitted to the VMTH of the FVM between January 2006 and July 2016, 179 had a clinical diagnosis of abomasum disease. Only 24 calves met the 3 additional inclusion criteria. In calves <3 months of age, the prevalence of abomasitis diagnosed clinically at the VMTH was 1.3% (24/1801).

Of the 24 calves with a clinical diagnosis of abomasitis, 1 was excluded for abomasal volvulus. Ultimately, 23 calves were included in the study (Data S2).

### 3.1 Signalment

Most of the calves were milk‐fed (23/23) females (21/23). There were 21 Holstein calves, 1 Blonde d'Aquitaine calf, and 1 calf of an undetermined breed. The average age at admission was 9.3 days (SD, 14.2; median, 3.0 days; range, 0‐62 days).

During the same period (January 1, 2006 to July 31, 2016), 75.2% (1355/1801) of the calves <3 months of age admitted to the VMTH were females. Regarding breed, 86.2% (1552/1801) were Holstein calves, 0.2% (3/1801) Blonde d'Aquitaine calves, 0.5% (9/1801) calves of an undetermined breed, and 13.2% (237/1801) calves of other breeds. The average age at admission was 24.1 days (SD, 27.3; median, 13 days; range, 0‐93 days).

### 3.2 History

The medical files mentioned ≥1 reason for presentation: bloating (9/23), generalized weakness (5/23), anorexia (5/23), colic (4/23), diarrhea (3/23), torticollis (1/23), lameness (1/23), birth at the VMTH (1/23), and undetermined (5/23). The duration of the clinical signs before admission was <24 hours (13/23), 24 hours (2/23), 48 hours (6/23), or 96 hours (2/23).

### 3.3 Clinical observations upon admission

Table 1 summarizes the values obtained for vital signs.^22^ If available, clinical signs observed were anorexia (13/14), positive succussion (13/14), lassitude (16/19), decubitus (15/18), abnormal mucosal color (injected, pale, cyanosis; 13/17), abnormal feces (diarrhea or blood; 10/14), lack of sucking reflex (8/13), presence of a ping (6/13), and colic (4/12). The percentages of dehydration were <5% (2/17), 5%‐7% (7/17), 7%‐10% (5/17), and 10%‐12% (3/17).

**Table 1:** Summary of the available vital variables on admission of the calves included in this study

Variables	Units	Mean [+/− SD]	Median [range]	Reference values^a^	Effective (%)		
					Decreased	Normal	Increased
Rectal temperature	°C	38.0 [+/− 2.2]	38.6 [32.6; 41.1]	[38.5; 39.5]	10 (46%)	6 (27%)	6 (27%)
Heart rate	bpm	154 [+/− 40]	148 [80; 240]	[90; 120]	1 (5%)	4 (18%)	17 (77%)
Respiratory rate	rpm	63 [+/− 27]	58 [24; 128]	[20; 50]	0 (0%)	9 (41%)	13 (59%)

a The reference values were obtained from the literature.^22^

### 3.4 Clinical pathology on admission

Tables 2 and 3 summarize the available CBC and SBP results. In addition, immature neutrophils (15/18) and toxic changes in the neutrophilic lineage (12/18) were observed on the blood smear.

**Table 2:** Summary of the available hematologic variables obtained within the first 24 hours after admission of the calves included in this study

Variables	Units	Mean [+/− SD]	Median [range]	Reference values^a^	Effective (%)		
					Decreased	Normal	Increased
PCV	%	35 [+/− 9.9]	33 [22; 53]	[26; 40]	5 (23%)	11 (50%)	6 (27%)
Total solids	g/dL	6.2 [+/− 0.76]	6.2 [5.0; 8.5]	[6.8; 8.6]	19 (86%)	3 (14%)	0 (0%)
Fibrinogen	mg/dL	422 [+/− 207]	350 [200; 900]	[200; 500]	0 (0%)	14 (78%)	4 (22%)
Total Leukocytes	Per μL	15 064 [+/− 11 567]	10 500 [3350; 48 380]	[6200; 13 600]	4 (22%)	6 (33%)	8 (45%)
Segmented neutrophils	Per μL	10 278 [+/− 10 017]	7210 [1260; 39 190]	[1100; 3600]	0 (0%)	6 (33%)	12 (67%)

a The reference values were obtained from the Diagnostic Service of the Faculty of Veterinary Medicine (Saint‐Hyacinthe, Canada).

**Table 3:** Summary of the available biochemical variables (serum) obtained within the first 24 hours after admission of the calves included in this study

Variables	Units	Mean [+/− SD]	Median [range]	Reference values^a^	Effective (%)		
					Decreased	Normal	Increased
pH	‐	7.28 [+/− 0.20]	7.34 [6.84; 7.50]	[7.35; 7.45]	8 (50%)	6 (37%)	2 (13%)
Sodium ion	mEq/L	135 [+/− 6.6]	137 [120; 143]	[134; 147]	4 (19%)	17 (81%)	0 (0%)
Potassium ion	mEq/L	5.5 [+/− 1.5]	5.2 [3.0; 8.3]	[3.86; 5.28]	2 (9%)	9 (43%)	10 (48%)
Chloride ions	mEq/L	92.7 [+/− 8.9]	93.0 [77.8; 106]	[96.4; 109.2]	15 (71%)	6 (29%)	0 (0%)
Total CO_2_	mEq/L	23.9 [+/− 8.0]	23.8 [13.4; 40.2]	[22.0; 33.0]	8 (44%)	8 (44%)	2 (11%)
Glucose	mg/dL	112.0 [+/− 121]	85.4 [5.4; 595]	[46.9; 88.3]	3 (15%)	9 (45%)	8 (40%)
l‐lactate	mg/dL	72.4 [+/− 46.4]	62.2 [7.21; 146.8]	[<13.5]	N/A	1 (6%)	15 (94%)
Urea	mg/dL	23.9 [+/− 12.1]	22.1 [10.0; 52.1]	[4.5; 18.2]	0 (0%)	5 (36%)	9 (64%)
Creatinine	mg/dL	2.57 [+/− 1.71]	2.13 [0.97; 7.23]	[0.61; 1.49]	0 (0%)	4 (29%)	10 (71%)
γ‐Glutamyl‐transferase	U/L	890 [+/− 646]	1082 [38; 1838]	[9.6; 39]	0 (0%)	1 (7%)	13 (93%)
Glutamate dehydrogenase	U/L	12.2 [+/− 10.3]	10.6 [2.0; 33.0]	[3; 45]	2 (14%)	12 (86%)	0 (0%)

a The reference values were obtained from the Diagnostic Service of the Faculty of Veterinary Medicine (Saint‐Hyacinthe, Canada).

### 3.5 Treatment on admission

Two calves died at admission, and 1 was euthanized before starting treatments, which were initiated in the remaining cases (20/20). In decreasing order of frequency, treatments included IV fluid therapy (20/20), IV antibiotics (20/20), butorphanol (10/20), erythromycin (7/20; as a prokinetic modifier), ranitidine (7/20), lidocaine infusion (5/20), flunixin meglumine (4/20), sucralfate (1/20), pantoprazole (1/20), and omeprazole (1/20).

The following IV antimicrobials were used: sodium ampicillin (12/20), sodium ampicillin and trimethoprim‐sulfadoxine (TMS) (6/20), TMS (1/20), and sodium penicillin G (1/20). In 9 of 20 calves, >1 route of administration was chosen: PO procaine penicillin G (5/9), intra‐abomasal procaine penicillin G (4/9), intra‐abomasal sodium ampicillin (2/9), and intraperitoneal procaine penicillin G (1/9).

Information about the feeding program established during the hours after admission was available in 19 cases: fasting (15/19), milk replacer (3/19), and colostrum (1/19). Of the 12 deceased calves, 10 died before their first meal. Before dying, 1 calf had been fasted, and 1 had been tube fed with colostrum. The term progressive feeding was mentioned in 9 of the 11 surviving calves' feeding programs. When information was available (10/11), all feeding programs of the surviving calves (10/10) included at least 3 milk meals per day. Similarly, when information was available (6/11), the first meals were 250 mL, 500 mL, or 1 L for 3, 1, and 2 calves, respectively.

Abomasal decompression was effective: 3 times for the 3 surgical interventions, twice for both transabdominal punctures, and once for the 5 esophageal intubations. Six right flank laparotomies were performed. These surgical interventions included a cecum decompression using a needle, or typhlotomy (5/6); an abomasal decompression using a needle, or abomasotomy (3/6); an intra‐abomasal antibiotic injection (2/6); and an enterectomy with laterolateral anastomosis (1/6). This last calf was concurrently diagnosed with atresia coli and abomasitis. When reported (5/6), surgical findings indicated abnormal abomasal content (severe gas or liquid distension or both) in 3 calves (3/5) and an abnormal abomasal wall (thickness, emphysema, hemorrhage, or necrosis) in 3 calves (3/5).

### 3.6 Necropsy findings

Of the 12 deceased calves, 11 were necropsied. Abnormal abomasal content was observed in all 10 necropsied calves for which information was available: increased quantity (9/10), reddish or brownish color (5/10), stench (3/10), and presence of blood clots (2/10). The wall of the abomasum was considered abnormal in all 11 necropsied calves: abnormal color (9/11), edema (7/11), emphysema (6/11), ulcer (5/11), thickened (4/11), necrosis (3/11), and hemorrhage (1/11). Four calves (4/5) had multiple, confluent, nonperforated abomasal ulcers. One calf (1/5) had a perforated abomasal ulcer. Three calves (3/11) had macroscopic lesions limited to the abomasum. The other 8 calves had other organs involved at the same time: intestinal tract (8/8), lungs (6/8), omasum (2/8), heart (1/8), and rumen (1/8).

The histopathologic findings of the abomasum included mucosal lesions in 9 of the 11 necropsied calves: hemorrhage (7/9), necrosis (5/9), congestion (3/9), inflammation (2/9), edema (2/9), and fibrin, thrombi, emphysema, ulcer, mineralization, and pustule (1/9). Submucosal lesions were observed in 10 of the 11 necropsied calves: edema (7/10), emphysema (6/10), inflammation (6/10), hemorrhage (5/10), fibrin (4/10), and congestion (1/10). Abnormal findings of the serosa were observed only in 2 calves: fibrin (1/2) and congestion (1/2). Bacteria could be visualized in the abomasal wall in 7 of 11 calves: gram‐positive rods (4/7), gram‐positive cocci (4/7), and mixed undetermined populations (1/7).

### 3.7 Bacteriology

Table 4 summarizes the results of bacteriological culture. Abomasal samples were taken from 11 calves for bacteriological culture (11/23). The antemortem samples were abomasal content (5/6) or abomasal wall (1/6). These samples were taken by transabdominal ultrasound‐guided puncture (2/6), by orogastric tube (2/6), by unspecified methods (1/6), or by partial abomasal excision during surgery (1/6). The nature of all the postmortem samples was not mentioned. *C. perfringens* was isolated and identified mainly from anaerobic cultures of postmortem samples (6/7). In our study, *Clostridium novyi*, *Clostridium septicum*, and *Clostridium chauvoei* were never isolated and identified by immunofluorescence on the postmortem samples (0/6). Although the aerobic cultures isolated and identified *E. coli* in 7 of the 8 abomasal postmortem samples, only 2 *E. coli* were identified with a virotype of interest.

**Table 4:** Summary of the bacteriological culture targeted for *Clostridium* spp. and *Escherichia coli* and obtained from abomasal samples

Cases identification		Aerobic culture		Anaerobic	*E. coli*	*Clostridium perfringens* type A (toxin)		*Clostridium septicum*, *Clostridium novyi*,
		*Clostridium* spp.	*E. coli*	culture *C. perfringens*	Target culture and virotyping (PCR)	PCR	ELISA	*Clostridium chauvoei* IF
Antemortem samples	1	*UR*	*UR*	Pos.	P, STa, Aero, Tsh	*UR*	*UR*	*UR*
	2	*UR*	*UR*	Neg.	Neg.	*UR*	*UR*	*UR*
	3	Neg.	Pos.	Neg.	*UR*	*UR*	*UR*	*UR*
	4	*UR*	*UR*	Neg.	*UR*	*UR*	*UR*	*UR*
	5	*UR*	*UR*	Pos.	*UR*	*UR*	*UR*	*UR*
	6	*UR*	*UR*	Pos.	*UR*	*UR*	*UR*	*UR*
	Subtotal	0/1	1/1	3/6				
Postmortem samples	4	Neg.	Neg.	Neg.	*UR*	*UR*	*UR*	Neg.
	5	Pos.	Pos.	Pos.	*UR*	*UR*	*UR*	Neg.
	6	Pos.	Pos.	Pos.	Stx2	*UR*	Pos.	Neg.
	7	Neg.	Pos.	Pos.	*UR*	*UR*	*UR*	Neg.
	8	Pos.	Pos.	Pos.	*UR*	*UR*	*UR*	*UR*
	9	Neg.	Pos.	Pos.	*UR*	*UR*	*UR*	Neg.
	10	Neg.	Pos.	Pos.	*UR*	Pos.	*UR*	*UR*
	11	Pos.	Pos.	*UR*	Stx1/Stx2	*UR*	*UR*	Neg.
	Subtotal	4/8	7/8	6/7		2/2		0/6
Total		4/9	8/9	9/13		2/2		0/6

Abbreviations: Aero, aerobactin; IF, immunofluorescence; Neg., negative result; P, fimbriae P; PCR, polymerase chain reaction; Pos., positive result; STa, thermostable enterotoxin; Stx, Shiga toxin; Tsh, temperature‐sensitive hemagglutinin; *UR*, unrealized test.

### 3.8 Short‐term case‐fatality rate

Of the 23 calves included in the study, 12 died during hospitalization. Of those 12 calves, 7 were euthanized. It was not possible to find in the medical files whether euthanasia was related to the cost of treatment or poor prognosis. The short‐term case‐fatality rate was 52% (12/23).

**Figure 1:**
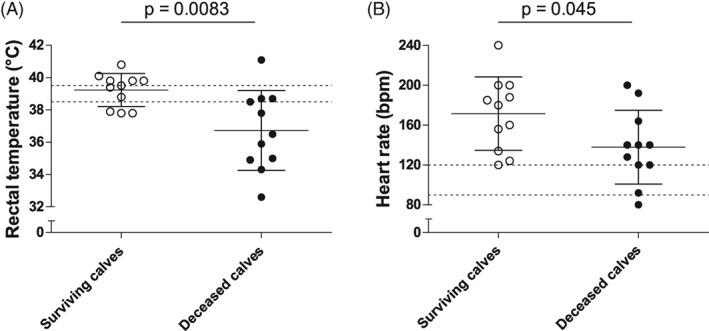
Summary of the univariate analysis of the different parameters that were significantly different between calves with positive or negative outcomes based on the short‐term outcome. Vertical scatter plot representation of the available admission values of the vital parameters that were significantly different between the surviving (white) and the deceased (black) calves: rectal temperature (A, n = 22) and heart rate (B, n = 22). Individual values, means, SDs, and reference ranges are shown

**Figure 2:**
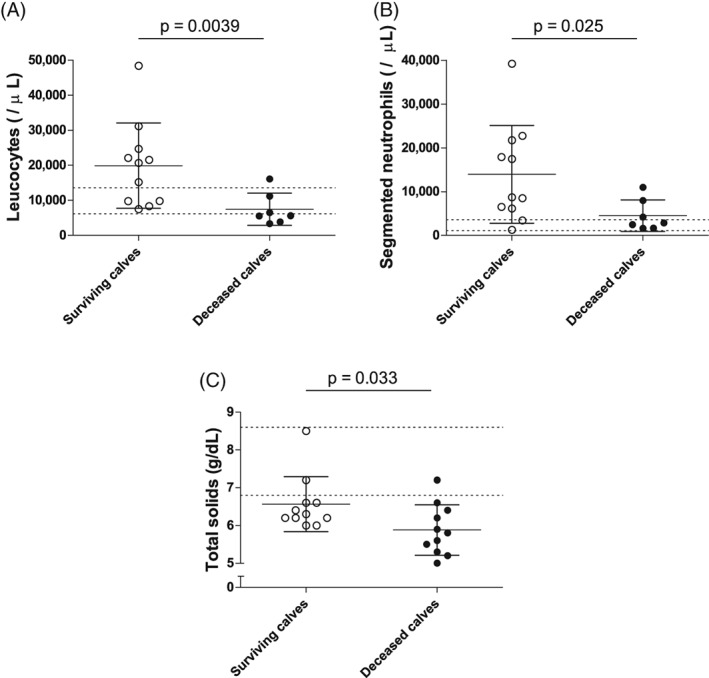
Summary of the univariate analysis of the different parameters that were significantly different between calves with positive or negative outcomes based on the short‐term outcome. Vertical scatter plot representation of the available admission values of the hematologic parameters that were significantly different between the surviving (white) and the deceased (black) calves: leucocytes (A, n = 18), segmented neutrophils (B, n = 18), and total solids (C, n = 22), Individual values, means, SDs, and reference ranges are shown

**Figure 3:**
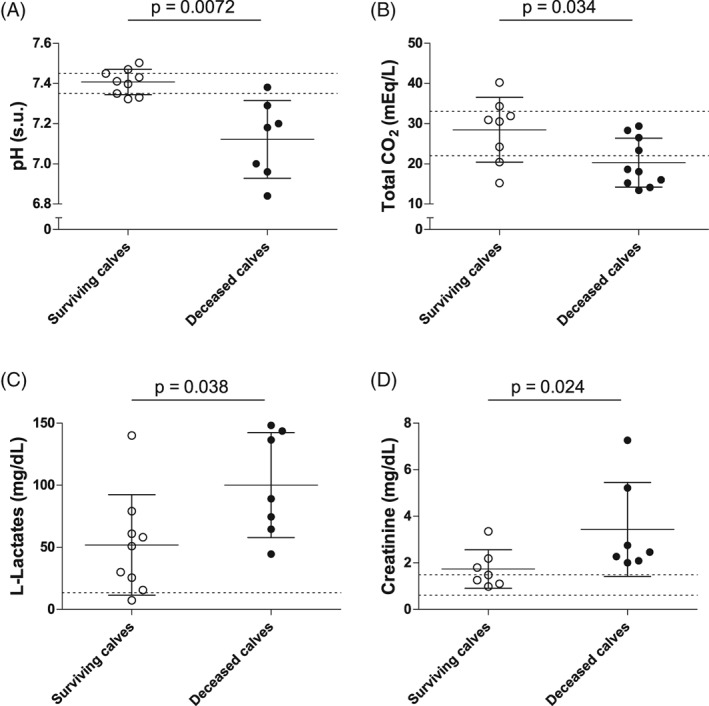
Summary of the univariate analysis of the different parameters that were significantly different between calves with positive or negative outcomes based on the short‐term outcome. Vertical scatter plot representation of the available admission values of the biochemical parameters (serum) that were significantly different between the surviving (white) and the deceased (black) calves: pH (A, n = 16), total CO_2_ (B, n = 18), l‐lactates (C, n = 16), and creatinine (D, n = 14). Individual values, means, SDs, and reference ranges are shown

Based on the short‐term issues, the fatal cases were significantly more hypothermic (*P* = .0083) and less tachycardic (*P* = .045) than surviving calves. The fatal cases had blood pH (*P* = .0072), TCO_2_ concentration (*P* = .034), total plasma solids (*P* = .033), leukocyte counts (*P* = .0039), and neutrophil counts (*P* = .025) that were significantly lower than those of the surviving calves. The fatal cases also had l‐lactate (*P* = .0038) and creatinine (*P* = .024) concentrations that were significantly higher than those of the surviving calves (Figures 1, 2, 3).

### 3.9 Long‐term case‐fatality rate

Mean time (with SD) and median time (with minimum and maximum) between discharge and telephone follow‐up were 4.8 (2.1) and 4.7 (1.6; 9.4) years, respectively. Of the 11 calves discharged from the VMTH, 2 calves died of unknown causes in the months after their discharge and 2 calves were lost to follow‐up. The long‐term case‐fatality rate was 67% (14/21).

Based on the long‐term issues, the fatal cases were significantly more hypothermic (*P* = .044) than the surviving calves. The fatal cases had blood pH (*P* = .0056) and TCO_2_ concentrations (*P* = .014) significantly lower than those of the surviving calves. The fatal cases also had l‐lactate (*P* = .0082) and creatinine (*P* = .026) concentrations significantly higher than those of the surviving calves. In the long‐term, the fatal cases spent significantly less time in the hospital than did the surviving calves (*P* = .0045).

## 4 DISCUSSION

To date, most clinical data describing abomasitis in calves consisted of postmortem observations. In our retrospective observational study, we confirmed and described the clinical signs and provided additional results from CBC and SBP, consistent with our hypotheses of sepsis and upper gastrointestinal stasis. Short‐term and long‐term case‐fatality rates were 52 and 67%, respectively.

In our study, we regarded abomasitis as a syndrome (ie, an association of clinical signs to guide the diagnosis, without predicting the underlying etiology). We could not differentiate *C. perfringens* type A abomasitis from *E. coli* abomasitis. We could not differentiate abomasitis with abomasal ulcers from abomasitis without abomasal ulcers. Indeed, abomasal ulcers were reported at necropsy in 5 calves, without antemortem suspicion. Similarly, on clinical examination, splashes or gas sounds could have misled the clinicians: abomasitis, left abomasal displacement, or abomasal volvulus can be responsible for these clinical observations. However, if we considered necropsy and histology as the reference tests, the decision to select cases based on clinical diagnoses was accurate because all necropsied cases were confirmed as true cases of abomasitis.

The clinical presentation of calves in our study was similar to previously published findings: acute clinical onset, accompanied by abdominal distention, anorexia, dehydration, positive succussion, lassitude, colic, diarrhea, and systemic shock.^2^, ^4^, ^6^, ^7^, ^8^, ^10^, ^11^, ^12^, ^14^, ^16^, ^17^, ^18^, ^23^, ^24^, ^25^ The findings that seem to be more common in our study are tachycardia, tachypnea, and hypothermia.

These results supported our hypotheses. Changes in vital signs (rectal temperature, heart rate, and respiratory rate) that were associated with hematological changes, bacteriological culture results, and multisystemic lesions on necropsy were suggestive of sepsis.^26^ The SBP changes could be explained by the hemodynamic changes associated with severe abomasitis and its consequences: hyper‐l‐lactatemia and azotemia. The positive succussion and the hypochloremia without hyponatremia, associated with abomasal dilatation (inclusion criterion), suggested upper gastrointestinal stasis, including abomasal stasis. To date, the only alterations mentioned in the veterinary literature were hyperglycemia^8^ and metabolic acidosis.^11^ In this study, the biochemical reference ranges used were obtained from the Diagnostic Service of the FMV. We noted hyperglycemia (>88.3 mg/dL) in 8 of the 20 calves for which this information was available. Our interpretation would be different if the reference intervals for calves reported in the literature (approximately 90‐126 mg/dL)^27^ were used. Using this reference range, 12 of 20 calves would have been considered hypoglycemic (<90 mg/dL) and 3 calves hyperglycemic (>126 mg/dL). Based on TCO_2_ measurement, metabolic acidosis was suspected in 8 of the 18 for which this information was available. Total CO_2_ is an estimate of bicarbonates (${\mathrm{HCO}}_{3}^{-}$), and mixed acid base disturbances may be difficult to assess based on this individual venous result.

In addition to the biases inherent to a retrospective study, such as missing data and absence of follow‐up examinations, a selection bias was associated with the study population. The calves were treated at a second‐level VMTH, to which animals of high value or animals requiring intensive medical care are referred. Moreover, most of the cattle referred to the VMTH of the FMV are dairy breeds, mainly Holstein. Because of this selection bias, our results should be extrapolated with caution to other populations. To minimize selection bias associated with the terms used to describe these calves, our inclusion criteria were based only on history and clinical observations. The cost of treatment and poor outcome reported in the current veterinary literature could have influenced medical euthanasia decisions, which represents a bias that could have worsened the prognosis observed in this study. Because of the small number of calves included, study power was limited, and additional associations could have been found between outcome and the variables studied. Only descriptive analysis was performed, and we did not correct for confounding variables or interactions.

The prognosis of abomasitis is reported to be poor.^2^, ^5^, ^6^, ^7^, ^8^, ^9^, ^10^, ^11^, ^12^, ^14^, ^15^, ^16^, ^17^, ^18^ In this study, the case‐fatality rates were 52%‐67% and agreed with the hypothesis of a guarded prognosis. The variables associated with the fatal outcome of some calves are considered in relation to their biological interpretations: septic or hypovolemic shock.^26^ In our study, the lower heart rate was difficult to interpret. Hypothermia, decompensated shock, metabolic disorders, sepsis‐induced cardiomyopathy or some combination of these are possible explanations for this unusual finding.^28^, ^29^, ^30^, ^31^, ^32^ However, because of the limitations mentioned previously, conclusions should be drawn with caution.

Although most of the treatments provided included fluid treatment and systemic administration of antibiotics, sample sizes, clinical differences, and therapy variability did not allow us to adequately interpret the effectiveness of the therapeutic approaches and to make recommendations. Our clinical and paraclinical data suggested that treatments targeting shock also prevented pain and bacterial proliferation, although we doubted the efficiency of some treatments (eg, PO procaine penicillin G).

Finally, our bacteriological results must be evaluated cautiously because some samples were collected postmortem. Indeed, unlike antemortem samples, abomasal samples collected during necropsy indicated invasion by commensal flora. On histology, the observation of intralesional bacteria confirmed their potential pathogenetic role. The gram‐positive rods that were observed could represent *C. perfringens*. However, the isolation of *C. perfringens* alone did not confirm its etiologic role. In addition, the presence of the toxin is necessary,^26^ but in our study (Table 4), clostridial toxins were identified in only 2 samples.

In conclusion, in this study from our referral center, the clinical presentation of calves with abomasitis syndrome might reflect sepsis. We identified some variables (rectal temperature, blood pH, total CO_2_ concentration, l‐lactate, and creatinine) associated with the short‐ and long‐term outcomes of these calves. Further prospective studies are essential to determine predisposing factors (eg, quantity and quality of milk meals and esophageal tube feeding) to assess the suspected bacterial etiology (eg, *C. perfringens* type A, *E. coli*, or *Sarcina* spp.) and to identify prognostic factors or efficient therapeutic approaches in the general cattle population.

## CONFLICT OF INTEREST DECLARATION

A fund from Zoetis for the clinical research; Zoetis was not involved in the study design, data collection, data analysis, and writing of the manuscript. Authors declare no conflict of interest.

## OFF‐LABEL ANTIMICROBIAL DECLARATION

Unlikely in the Canadian legislation, the authors declare the off‐label use of TMS in the American legislation.

## INSTITUTIONAL ANIMAL CARE AND USE COMMITTEE (IACUC) OR OTHER APPROVAL DECLARATION

Authors declare no IACUC or other approval was needed.

## HUMAN ETHICS APPROVAL DECLARATION

Authors declare human ethics approval was not needed for this study.

## Supporting information


**Data S1** STROBE Vet‐Statement—Checklist (20‐21)

**Data S2** Flow diagram
