# Supplementary material for: Abomasitis in calves: A retrospective cohort study of 23 cases (2006‐2016)
Source: J Vet Intern Med. 2020 Feb 14;34(2):1018–27. doi: 10.1111/jvim.15726 (PMC7096666; doi:10.1111/jvim.15726)
Supplement: Supplementary file 1 — Data S1 STROBE Vet‐Statement—Checklist (20‐21) [file JVIM-34-1018-s001.pdf]

	Item No	Recommendation	Page No
**Title and abstract**	1	(*a*) Indicate the study's design with a commonly used term in the title or the abstract	1
		(*b*) Provide in the abstract an informative and balanced summary of what was done and what was found: why the study was conducted, the design, the results, the limitations, and the relevance of the findings.	4
**Introduction**			
Background/rationale	2	Explain the scientific background and rationale for the investigation being reported	6
Objectives	3	*(a)* State specific objectives, including any prespecified hypotheses	7
		(b) Ensure that the level of organization is clear for each objective and hypothesis	7
**Methods**			
Study design	4	Present key elements of study design early in the paper	8
Setting	5	*(a)* Describe the setting, locations, and relevant dates, including periods of recruitment, exposure, follow-up, and data collection	8
		(b) If applicable, include information at each level of organization.	-
Participants	6	(*a*) Give the eligibility criteria, and the sources and methods of selection of participants. Describe methods of follow-up	8
		(*b*) For matched studies, give matching criteria and number of exposed and unexposed	-
Variables	7	*(a)* Clearly define all outcomes, exposures, predictors, potential confounders, and effect modifiers. Give diagnostic criteria, if applicable	8
		(*b*) Describe the level of organization at which each variable was measured	8-10
		(c) For hypothesis-driven studies, the putative causal structure among variables should be described	-
Data sources/ measurement	8*	*(a)* For each variable of interest, give sources of data and details of methods of assessment (measurement). Describe comparability of assessment methods if there is more than one group	8-10
		(b) If a questionnaire was used to collect data, describe its development,	

	Item No	Recommendation	Page No
		validation, and administration	9
		(c) Describe whether or not individuals involved in data collection were blinded, when applicable	-
		(d) Describe any efforts to assess the accuracy of the data (including methods used for "data cleaning" in primary research, or methods used for validating secondary data)	-
Bias	9	Describe any efforts to address potential sources of bias	17-18
Study size	10	*(a)* Describe how the study size was arrived at for each relevant level of organization	8 &11
		(b) Describe how nonindependence of measurements was incorporated into sample-size considerations, if applicable	-
		(c) If a formal sample-size calculation was used, describe the parameters, assumptions, and methods that were used, including a justification for the effect size selected	-
Quantitative variables	11	Explain how quantitative variables were handled in the analyses. If applicable, describe which groupings were chosen and why	7-8
Statistical methods	12	(*a*) Describe all statistical methods, including those used to control for confounding	9-10 & 17-18
		(*b*) Describe any methods used to examine subgroups and interactions	-
		(*c*) Explain how missing data were addressed	10
		(*d*) If applicable, describe the analytical approach to loss to follow-up, matching, complex sampling, and multiplicity of analyses	9-10
		(*e*) Describe any methods used to assess the robustness of the analyses	-
**Results**			
Participants	13	*(a)* Report numbers of individuals at each stage of study—eg numbers potentially eligible, examined for eligibility, confirmed eligible, included in the study, completing follow-up, and analysed	11
		(b) Give reasons for non-participation at each stage	-
		(c) Consider use of a flow diagram	Supp2
Descriptive data	14	*(a)* Give characteristics of study participants (eg demographic, clinical,	11

	Item No	Recommendation	Page No
		social) and information on exposures and potential confounders	
		(b) Indicate number of participants with missing data for each variable of interest	11-15
		(c) Summarise follow-up time (eg, average and total amount)	15
Outcome data	15	*(a)* Report outcomes as appropriate for the study design and summarize at all relevant levels of organization	14-15
		(b) For proportions and rates, report the numerator and denominator	15
		(c) For continuous outcomes, report the number of observations and a measure of variability	-
Main results	16	(*a*) Give unadjusted estimates and, if applicable, confounder-adjusted estimates and their precision (eg, 95% confidence interval). Make clear which confounders were adjusted for and why they were included	11-15 & 17-18
		(*b*) Report category boundaries when continuous variables were categorized	-
		(*c*) If relevant, consider translating estimates of relative risk into absolute risk for a meaningful time period	-
Other analyses	17	Report other analyses done—eg analyses of subgroups and interactions, and sensitivity analyses	15
**Discussion**			
Key results	18	Summarise key results with reference to study objectives	16-17
Limitations	19	Discuss limitations of the study, taking into account sources of potential bias or imprecision. Discuss both direction and magnitude of any potential bias	17-18
Interpretation	20	Give a cautious overall interpretation of results considering objectives, limitations, multiplicity of analyses, results from similar studies, and other relevant evidence	16-18
Generalisability	21	Discuss the generalisability (external validity) of the study results	17-18
**Other information**			
Transparency	22	*(a)* Funding—Give the source of funding and the role of the funders for the present study and, if applicable, for the original study on which the present article is based	1
		(b) Conflicts of interest—Describe any conflicts of interest, or lack thereof,	1

Item No	Recommendation	Page No
	for each author	
	(c) Describe the authors’ roles—Provision of an author’s declaration of transparency is recommended	1
	(d) Ethical approval—Include information on ethical approval for use of animal and human subjects	1
	(e) Quality standards—Describe any quality standards used in the conduct of the research	1

Supporting Information 1: STROBE Vet-Statement—Checklist (20-21)
