# Supplementary figures and images for: Abomasitis in calves: A retrospective cohort study of 23 cases (2006‐2016)
Source: J Vet Intern Med. 2020 Feb 14;34(2):1018–27. doi: 10.1111/jvim.15726 (PMC7096666; doi:10.1111/jvim.15726)

Screening

Eligibility

Inclusion

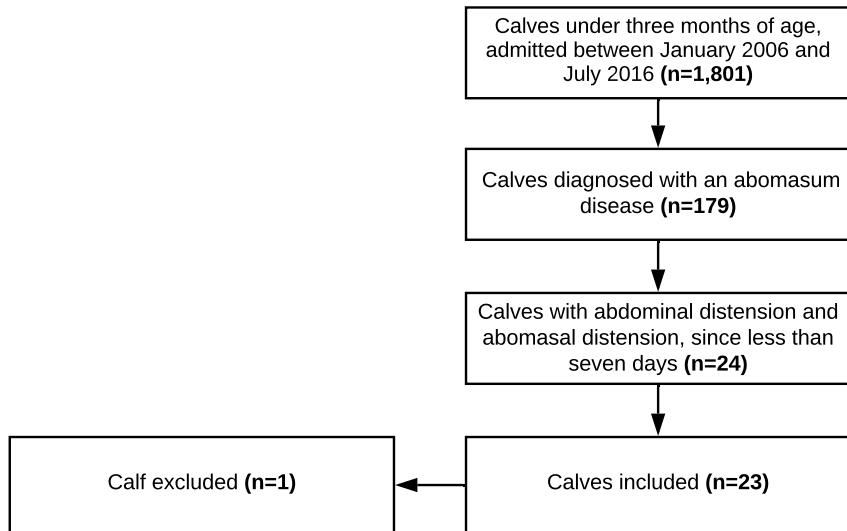

**Supporting information 2:** Flow diagram

Supplement: Supplementary file 2 — Data S2 Flow diagram [file JVIM-34-1018-s002.pdf]
